# Uncovering the regeneration strategies of zebrafish organs: a comprehensive systems biology study on heart, cerebellum, fin, and retina regeneration

**DOI:** 10.1186/s12918-018-0544-3

**Published:** 2018-03-19

**Authors:** Fang-Yu Liu, Te-Cheng Hsu, Patrick Choong, Min-Hsuan Lin, Yung-Jen Chuang, Bor-Sen Chen, Che Lin

**Affiliations:** 1 Department of Electrical Engineering, National Tsing Hua University, Hsinchu, 30013 Taiwan; 2 Department of Medical Science and Institute of Bioinformatics and Structural Biology, National Tsing Hua University, Hsinchu, 30013 Taiwan

**Keywords:** System biology, Zebrafish, Regeneration, Heart, Fin, Retina, Cerebellum, PPI (protein-protein interactions)

## Abstract

**Background:**

Regeneration is an important biological process for the restoration of organ mass, structure, and function after damage, and involves complex bio-physiological mechanisms including cell differentiation and immune responses. We constructed four regenerative protein-protein interaction (PPI) networks using dynamic models and AIC (Akaike’s Information Criterion), based on time-course microarray data from the regeneration of four *zebrafish* organs: heart, cerebellum, fin, and retina. We extracted core and organ-specific proteins, and proposed a recalled-blastema-like formation model to uncover regeneration strategies in *zebrafish*.

**Results:**

It was observed that the core proteins were involved in TGF-β signaling for each step in the recalled-blastema-like formation model and TGF-β signaling may be vital for regeneration. Integrins, FGF, and PDGF accelerate hemostasis during heart injury, while Bdnf shields retinal neurons from secondary damage and augments survival during the injury response. Wnt signaling mediates the growth and differentiation of cerebellum and fin neural stem cells, potentially providing a signal to trigger differentiation.

**Conclusion:**

Through our analysis of all four *zebrafish* regenerative PPI networks, we provide insights that uncover the underlying strategies of *zebrafish* organ regeneration.

## Background

Regeneration processes orchestrate various bio-physiological mechanisms for wound healing such as the immune response, cell proliferation, and differentiation. In mammals, the regenerative capacity of organs, such as the central nervous system (CNS, including cerebellum and cordial spinal), peripheral nervous system (PNS), heart, and limbs, is generally limited. This makes it difficult for mammals to recover from damages, such as heart defects and traumatic cerebellum injuries. Understanding the cellular and molecular mechanisms behind the regenerative abilities of these organs may bring about great improvements in regenerative medicine. Therefore, three broad questions for investigating regenerative mechanisms were proposed: (i) what is the vital signal needed to carry out a regenerative response drive, (ii) what triggers the production of this signal, and (iii) how are the differentiated tissue cells patterned into the correct structures [1]? Since various bio-physiological processes are conducted in the regeneration process, it is reasonable to assume that there exists some common, vital signal needed to carry out a regenerative response drive in the regeneration of different organs. Consequently, it is important that studies compare the regeneration of different organs to answer these questions.

Recently, the main focus of regenerative research has been on identifying the source of cell proliferation for repairing lost tissue. In cerebellum regeneration, there are neural stem cells niches along the whole rostro-caudal cerebellum axis that support cerebellum injury repair [2, 3]. In retina regeneration, Müller glia de-differentiate and re-enter the cell cycle to produce to regenerate retinal neuron including progenitor cells, which act as the source of photoreceptor cells [4]. The majority of the regenerating myocardium is believed to derive from resident Cmlc2 cardiomyocytes [5]. These regeneration processes control similar biological functions, including assembling resident cells to act as the primary source of organ regeneration. This seems to indicate that a common regenerative mechanism may exist throughout different organs and tissues [1].

While several animal models have been proposed for investigation into the underlying mechanism of such organ regeneration, *zebrafish* are one of the more attractive candidates due to their strong regenerative ability. Specifically, *zebrafish* are capable of regenerating a wide range of organs including heart, fins, CNS, jaw, (lateral-line) hair cells, pancreas, liver, and kidneys [6]. In addition to their strong regenerative ability, *zebrafish* have also been considered a practical model organism due to the similarity of their genome to that of humans, their high reproductive rates, and low maintenance costs.

In this study, we attempted to integrate existing research to investigate the mechanism underlying the regeneration of different organs through a comprehensive systems biology approach focused on *zebrafish* heart, cerebellum, fin, and retina. Systems biology theories and approaches, such as protein-protein interaction (PPI) network construction and dynamic modeling, are useful for investigation into such common regenerative mechanisms. In the present study, we constructed candidate PPI networks using microarray data from the regeneration experiments of four *zebrafish* organs including heart, cerebellum, fin, and retina. Next, we used dynamic modeling and model order selection techniques to identify protein interaction ability to augment the PPIs. We integrated these data and techniques to construct four refined PPI networks for the regeneration of all four *zebrafish* organs. By comparing the four regenerative networks, we identified both core (common) and organ-specific proteins, which are useful in the elucidation of the common and organ-specific regeneration mechanisms of the *zebrafish*. With the help of integrated online tools, we conducted further pathway analysis to identify the key proteins and functional modules involved in the *zebrafish* regeneration process. To analyze the roles of significant proteins in the regeneration process and to investigate the underlying mechanisms of regeneration, we proposed a recalled-blastema-like formation model for further study. We hope that this work will assist in uncovering the regeneration strategies of *zebrafish* and provide a foundation for human regenerative medicine.

## Results

### Construction of zebrafish regenerative PPI networks

**Fig. 1:**
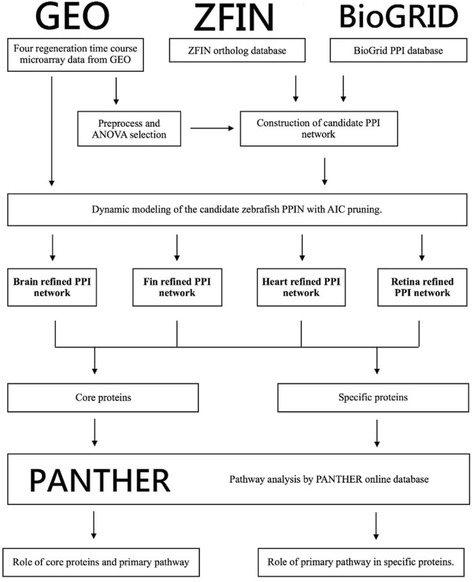
Flow chart of PPI network design and analysis approaches. This study used online datasets including the GEO online datasets, ZFIN ortholog database, BioGRID PPI database, and PANTHER protein classification system. Microarray data from four organ regeneration experiments including heart, cerebellum, fin, and retina, were used with high-throughput data and integrated dataset preprocessing to construct candidate PPI networks. Next, regression dynamic models were applied to derive the interaction abilities in each PPI, using the Akaike’s Information Criterion method to prune false-positive PPIs. Finally, dynamic PPI networks were refined for the four organ regeneration processes. Subsequent analysis of the intersection set (the core PPI network) and relative complement sets (the organ-specific proteins) to investigate the regeneration strategy for both the core PPI network and organ-specific proteins was conducted using PANTHER

To construct a regenerative PPI network, two steps are required: (i) Data selection, preprocessing and candidate network construction; and (ii) identification of PPI parameters and false positive PPI pruning using Akaike’s Information Criterion (AIC) [7]. A flowchart of the network construction process is depicted in Fig. 1.

In the first step, a candidate intracellular PPI network is obtained by collecting all intracellular protein interactions in *zebrafish*. The heart, cerebellum, fin, and retina time course microarray data sets were from Gene and Expression Omnibus (GEO) database of National Center of Biotechnology Information (NCBI) with accession number GSE56375 for cerebellum [8] with time points 0, 0.25, 1, and 3 days-post-injury (dpi), GSE37165 for fin [9] with time points 0, 0.5, 1, and 2 dpi, and GSE3303 for retina [8, 10] with time points 0, 2, 3, and 5 dpi. The heart microarray for time points 0, 0.25, 1, and 3 dpi was submitted to GEO with accession number GSE72348. The detailed protocol is provided in the Materials and Methods section. The cerebellum microarray data set was presented in a previous work from our group [8]. Handling of the animal model was approved by the Institutional Animal Care and Use Committee of National Tsing Hua University (IRB Approval No. 09808). For GSE56375 and GSE72348, 75-th percentile normalization was applied to both datasets. For GSE37165, GCRMA normalization was conducted. For GSE3303 all data were normalized and scaled by the Gene Chip Operating System software (version 1.3). One-way analysis of variance (ANOVA) was used to select differentially expressed genes with a *p*-value below 0.05 after Bonferroni correction [11, 12]. As a result, 26,568, 25,116, 7769 and 7630 proteins were obtained as the protein pools for the heart, cerebellum, fin, and retina regeneration processes, respectively.

Next, we examined potential interactions of *zebrafish* proteins in the selected protein pools using available PPI information. Since there were no existing *zebrafish* PPIs, human PPIs and ortholog information were used to obtain the candidate PPI network for *zebrafish* [13]. The human PPI data were extracted from Biological General Repository for Interaction Datasets (BioGRID, http://thebiogrid.org) while the ortholog data of *zebrafish* and *Homo sapiens* genes were extracted from the Zebrafish Model Organism Database (ZFIN, http://zfin.org) [13, 14]. The resulting candidate PPI networks of four *zebrafish* organs contained 3567 proteins and 7571 PPIs for heart, 3491 proteins and 6801 PPIs for cerebellum, 2075 proteins and 2635 PPIs for fin, as well as 2013 proteins and 2359 PPIs for retina, respectively. Since the candidate PPI network does not represent the actual intracellular protein interactions during the *zebrafish* regeneration process, further validation and pruning of the PPI network are necessary. The following dynamic model is used to describe intracellular protein interactions [15–18]:

1 $$ {x}_i\left[t+1\right]={x}_i\left[t\right]+{\sum}_{j=1}^{N_i}{a}_{ij}{x}_j\left[t\right]-{\lambda}_i{x}_i\left[t\right]+{k}_i+{\varepsilon}_i\left[t\right] $$

where *x*_*i*_[*t*] represents the protein expression level for the *i*-th *zebrafish* protein at time t, *N*_*i*_ represents corresponding number of PPIs for the *i*-th target protein, *λ*_*i*_ is the degradation effect for the *i*-th target protein, *x*_*j*_[*t*] denotes the protein expression level for the j-th *zebrafish* protein that can potentially interact with the *i*-th target protein, and *a*_*ij*_ denotes the corresponding interaction ability between the two proteins. The basal level is denoted by *k*_*i*_, and the stochastic noise due to model uncertainty or fluctuations in the microarray data is represented by *ε*_*i*_[*t*].

The interaction ability of a PPI network can be determined with the help of time-course microarray data. Although mRNA expression levels do not represent protein levels exactly but were shown to be positively correlated to protein levels [19, 20]. To identify the interaction ability, we first rewrite the dynamic eq. (1) into the following linear regression form:

2 $$ {\displaystyle \begin{array}{c}{\mathrm{x}}_{\mathrm{i}}\left[\mathrm{t}+1\right]=\left[\ {\mathrm{x}}_1\left[\mathrm{t}\right]\kern0.75em {\mathrm{x}}_2\left[\mathrm{t}\right]\kern0.5em \cdots \kern0.5em {\mathrm{x}}_{{\mathrm{N}}_{\mathrm{i}}}\left[\mathrm{t}\right]\kern0.75em {\mathrm{x}}_{\mathrm{i}}\left[\mathrm{t}\right]\kern0.75em 1\ \right]\left[\begin{array}{c}{\mathrm{a}}_{\mathrm{i}1}\\ {}{\mathrm{a}}_{\mathrm{i}2}\\ {}\vdots \\ {}{\mathrm{a}}_{{\mathrm{i}\mathrm{N}}_{\mathrm{i}}}\\ {}1-{\uplambda}_{\mathrm{i}}\\ {}{\mathrm{k}}_{\mathrm{i}}\end{array}\right]+{\upvarepsilon}_{\mathrm{i}}\left[\mathrm{t}\right]\\ {}={\upphi}_{\mathrm{i}}\left[\mathrm{t}\right]{\uptheta}_{\mathrm{i}}+{\upvarepsilon}_{\mathrm{i}}\left[\mathrm{t}\right]\end{array}} $$

where ϕ_i_[t] denotes the regression vector and θ_i_ denotes corresponding estimated parameter vector for the *i*-th target protein. We also interpolate additional time points within the microarray data by cubic spline method to avoid over-fitting. Equation (2) for different time points are listed as follows:

3 $$ {\displaystyle \begin{array}{c}{\mathrm{x}}_{\mathrm{i}}\left[{\mathrm{t}}_2\right]={\upphi}_{\mathrm{i}}\left[{\mathrm{t}}_1\right]{\uptheta}_{\mathrm{i}}+{\upvarepsilon}_{\mathrm{i}}\left[{\mathrm{t}}_1\right]\\ {}{\mathrm{x}}_{\mathrm{i}}\left[{\mathrm{t}}_3\right]={\upphi}_{\mathrm{i}}\left[{\mathrm{t}}_2\right]{\uptheta}_{\mathrm{i}}+{\upvarepsilon}_{\mathrm{i}}\left[{\mathrm{t}}_2\right]\\ {}\vdots \\ {}{\mathrm{x}}_{\mathrm{i}}\left[{\mathrm{t}}_{{\mathrm{M}}_{\mathrm{i}}}\right]={\upphi}_{\mathrm{i}}\left[{\mathrm{t}}_{{\mathrm{M}}_{\mathrm{i}}-1}\right]{\uptheta}_{\mathrm{i}}+{\upvarepsilon}_{\mathrm{i}}\left[{\mathrm{t}}_{{\mathrm{M}}_{\mathrm{i}}-1}\right]\end{array}} $$

where M_i_ denotes the number of time points after interpolating. Next, we rearrange eq. (3) as the following linear regression form:

4 $$ {\mathrm{X}}_{\mathrm{i}}={\Phi}_{\mathrm{i}}{\uptheta}_{\mathrm{i}}+{\mathrm{E}}_{\mathrm{i}} $$

where ${\mathrm{X}}_{\mathrm{i}}={\left[\ {\mathrm{x}}_{\mathrm{i}}\left[{\mathrm{t}}_2\right]\kern0.75em {\mathrm{x}}_{\mathrm{i}}\left[{\mathrm{t}}_3\right]\cdots {\mathrm{x}}_{\mathrm{i}}\left[{\mathrm{t}}_{{\mathrm{M}}_{\mathrm{i}}}\right]\kern0.5em \right]}^{\mathrm{t}}$, ${\Phi}_{\mathrm{i}}={\left[\ {\upphi}_{\mathrm{i}}\left[{\mathrm{t}}_1\right]\kern0.75em {\upphi}_{\mathrm{i}}\left[{\mathrm{t}}_2\right]\cdots {\upphi}_{\mathrm{i}}\left[{\mathrm{t}}_{{\mathrm{M}}_{\mathrm{i}}-1}\right]\ \right]}^{\mathrm{T}}$ and ${\mathrm{E}}_{\mathrm{i}}=\Big[\ {\upvarepsilon}_{\mathrm{i}}\left[{\mathrm{t}}_1\right]\kern0.5em {\upvarepsilon}_{\mathrm{i}}\left[{\mathrm{t}}_2\right]\cdots {\upvarepsilon}_{\mathrm{i}}\left[{\mathrm{t}}_{{\mathrm{M}}_{\mathrm{i}}-1}\right]$]. The parameter θ_i_ can be identified by solving the constrained least-squares minimization problem. Subsequently, Akaike’s information criterion (AIC) is applied to prune false-positive interactions. The formula of AIC is shown as follows:

5 $$ {\mathrm{K}}_{\mathrm{AIC}}\left({\mathrm{N}}_{\mathrm{i}}\right)=\log \left(\frac{1}{{\mathrm{M}}_{\mathrm{i}}}{\left({\mathrm{X}}_{\mathrm{i}}-\widehat{{\mathrm{X}}_{\mathrm{i}}}\right)}^{\mathrm{T}}\left({\mathrm{X}}_{\mathrm{i}}-\widehat{{\mathrm{X}}_{\mathrm{i}}}\right)\right)+\frac{2{\mathrm{N}}_{\mathrm{i}}}{{\mathrm{M}}_{\mathrm{i}}} $$

where M_i_ denotes the number of time points after interpolation, N_i_ denotes the number of identified parameter, $\widehat{{\mathrm{X}}_{\mathrm{i}}}$ denotes the estimated expression level. AIC includes both estimated residual error, $\frac{1}{{\mathrm{M}}_{\mathrm{i}}}{\left({\mathrm{X}}_{\mathrm{i}}-\widehat{{\mathrm{X}}_{\mathrm{i}}}\right)}^{\mathrm{T}}\left({\mathrm{X}}_{\mathrm{i}}-\widehat{{\mathrm{X}}_{\mathrm{i}}}\right)$, and model complexity, $\frac{2{\mathrm{N}}_{\mathrm{i}}}{{\mathrm{M}}_{\mathrm{i}}}$. Value of AIC increases as the number of parameters increases and decreases as the estimated residual error decreases. Nonetheless, increasing number of parameters may decrease estimated residual error. That is, there exists a tradeoff between model complexity and estimation accuracy. We solve for the minimum value of AIC to identify significant interactions through adjusting appropriate model order. By following these procedures, the refined zebrafish regenerative PPI network for heart, cerebellum, fin and retina were thus constructed. This procedure was applied to all four time-course *zebrafish* microarray data, which were obtained from corresponding regenerative experiments for heart, cerebellum, fin, and retina to construct the corresponding refined *zebrafish* regenerative PPI networks. Note that different platforms were used for the microarray data for the regeneration process for different organs. Although interaction identified in our PPI networks can be affected by the different normalization methods utilized by different platforms for our microarray data. However, whether the interaction is ‘present’ between two proteins is not affected by such normalization methods. In this study, we mainly focus on the comparison of regeneration PPI networks constructed for different organs. In our comparison, an interaction was considered to be common to both organ regenerations if this interaction was identified to be ‘present’ in both our constructed PPI networks. As a result, we were able to integrate data from different platforms for the comparison.

### Inspection of the constructed zebrafish regenerative PPI networks for heart, cerebellum, fin, and retina

After AIC model order detection, there were 2161 proteins (nodes) and 4517 PPIs (edges) in the constructed heart regeneration PPI network. The cerebellum regeneration PPI network contained 2074 proteins and 4102 PPIs; the fin regeneration PPI network contained 1085 proteins and 1841 PPIs. Finally, retina regeneration PPI network contained 945 proteins and 1434 PPIs (see S1 for the complete PPI network lists and figures). The top ten hub proteins of the four regenerative PPI networks, as ranked by the number of PPIs connecting them, are listed in Table 1 with their corresponding gene ontology (GO) function annotation. Several hub proteins were related to cell proliferation, cell cycle, and angiogenesis. For instance, H2afx, which regulates the G1-to-S-phase transition of the cell cycle, was identified to be a hub protein of the PPI networks of cerebellum, fin, and retina regeneration [21]. Another hub protein identified in all four regenerative PPI networks was Hdac1, which interacts with retinoblastoma tumor-suppressor protein, forming a complex that is key in the control of cell proliferation and differentiation [22]. Rb1 protein recruits chromatin-modifying enzymes and prevents the transcription of multiple cell cycle genes [23]. It is an inhibitor of the cell cycle and stabilizes constitutive heterochromatin to maintain the overall chromatin structure during regeneration [23]. The identification of these hub proteins partially validates our constructed PPI networks.

**Table 1:** The top 10 hub proteins ranked by number of PPIs (edges) and corresponding GO biological process in the regenerative PPI networks for heart, cerebellum, fin, and retina

Heart			Cerebellum			Fin			Retina		
Symbol	PPIs	Biological process	Symbol	PPIs	Biological process	Symbol	PPIs	Biological process	Symbol	PPIs	Biological process
Ubc	458		Ubc	346		Tp53	56	Apoptosis	Mycb	58	Sequence-specific DNA-binding transcription factor activity
Sumo2	105	Embryo development	Ywhaqb	70	Oxidoreductase activity	Tat	44	Cellular amino acid metabolism	EUr1	35	DNA-templated regulation of transcription
Mycb	90	Sequence-specific DNA binding Transcription factor activity	Tat	59	Cellular amino acid metabolism	Hdac1	40	Inhibition of cell proliferation	Tat	33	Cellular amino acid metabolism
Tp53	68	Apoptosis	Hdac1	55	Inhibition of cell proliferation	Sp1	39	Regulation of transcription from the RNA polymerase II promoter	Hdac1	33	Inhibition of cell proliferation
Ywhaqb	60	Oxidoreductase activity	Tk1	53	DNA biosynthesis	Esr1	35	DNA-templated regulation of Transcription	Src	30	Cell cycle
Hdac1	52	Inhibition of cell proliferation	Ar	48	DNA-templated regulation of transcription	Rb1	31	Regulation of cell cycle	Pou5f1	29	Embryonic pattern specification
Sp1	52	Regulation of transcription from the RNA polymerase II promoter	Smn1	47	Peripheral nervous system neuron Axonogenesis	H2afx	31	Cell cycle	H2afx	25	Cell cycle
Tgfbr1a	49	Protein phosphorylation	Mepce	47		Ywhag1	31	Cerebellum development	Sp1	24	Regulation of transcription from the RNA polymerase II promoter
Tat	49	Cellular amino acid metabolism	Ywhae1	46	Oxidoreductase activity	Smarca4	30	Cardiac muscle cell proliferation	Mdm2	23	Inhibition of apoptosis process
Yhl	49	Regulation of angiogenesis	H2afx	46	Cell cycle	Ncor1	29	Anterior/posterior pattern specification	Ncor1	23	DNA-templated regulation of transcription

The *zebrafish* regenerative PPI networks for heart, cerebellum, fin, and retina capture the differentially expressed proteins and their corresponding PPIs. Hub proteins have numerous PPIs and act as the bridges of the network. Therefore, the functions of these hub proteins may represent the primary characteristics of the PPI network. The GO biological processes of the hub proteins for the four regenerative networks include metabolism, regulation of the cell cycle, cell proliferation, pattern specification, apoptosis, and transcription. Grey shading represents the gene ontology of proteins related to proliferation and cell cycle, including Hadac1, H2afx, and Rb1 for cell cycle regulation and Smarca4 for cardiac muscle cell proliferation. It was observed that the regenerative PPI networks are closely related to cell cycle in the regeneration of all four organs

### Identification of core proteins, core PPI networks, and organ-specific proteins

Further analysis was performed to compare the four constructed regenerative PPI networks as follows: (i) identification of core proteins, which are a set of proteins obtained from overlaps across all four regenerative PPI networks, and (ii) further identification of core PPI networks, which correspond to those PPI networks associated with these core proteins in each organ regeneration network. As a result, 189 core proteins were observed in the four regenerative networks. Since the PPIs may be different in each regenerative network, there are four core PPI networks with the same nodes (core proteins) but different edges (PPIs). The number of edges of the four core PPI networks was 163 for heart, 178 for cerebellum, 176 for fin, and 162 for retina, respectively. The figures and complete lists for core networks are included and illustrated in S2 File and the GO biological processes of the 189 core proteins are recorded in S3 File. Since there might also exist specialized molecular functions during the regeneration process of different organs, organ-specific proteins were identified by taking the relative complement set of each regenerative PPI network with respect to other three PPI networks (see S4 File for a complete list of organ-specific proteins). In other words, an organ-specific protein is a unique protein that only exists in the regenerative PPI network of a particular organ.

### Pathway analysis for core and organ-specific proteins

The primary enriched pathways for core and organ-specific proteins were obtained by applying the online pathway classification tool, PANTHER, to the core and organ-specific proteins of the constructed *zebrafish* regenerative networks for heart, cerebellum, fin, and retina; and are presented in Table 2. The top three enriched pathways are shown in order with their corresponding proteins (see S5 File for complete pathway classification of both the core and organ-specific proteins). The primary pathways of the core proteins included the TGF-β signaling pathway, the gonadotropin releasing hormone receptor pathway, and the angiogenesis pathway. TGF-β plays an essential role in many cellular functions, including immunity, cancer, proliferation, and cellular differentiation. Several studies reported that TGF-β proteins were involved in *zebrafish* regenerative processes, including the cardiomyocyte proliferation in heart regeneration, the photoreceptor proliferative response in retinal regeneration, and the regulation of cell adhesion in fin regeneration [24, 25].

**Table 2:** Pathway distribution for core proteins and organ-specific proteins in the regeneration networks of the heart, cerebellum, fin, and retina

	Pathway	Proteins
Core proteins	TGF-β signaling pathway	Smad7, Jun, Mapk1, Mapk3, Map3k7, Smurf2, Smad3a, Skib, Smad2,Spaw
	Gonadotropin releasing hormone receptor pathway	Jun, Mapk1, Napk3, Cdc42, Map3k7, Pou2f1b, Smad3a, Map2k1,Smad2
	Angiogenesis	Crk, Jun, Mapk1, Mapk3, Pak2a, Pak1, Tcf7l2, Map2k1
Heart specific proteins	Gonadotropin releasing hormone receptor pathway	Fstb, Srf, Gata2b, Map2k2a, Pparaa, Prkcz, Bmpr2a, Slc2a1a, Fosb, 1hx2b, Pparg, Sos1, Tcf7, Sdf4
	Integrin signaling pathway	Parvaa, Rras, Map2k2a, Kras, Lamb1a, Rac3a, Tln1, Arl1, Mapk10, Vasp, Lama5, Sos1, Bcar1
	FGF signaling pathway	Ptpn11a, Map2k2a, Akt3a, Kras, Ppp2r2bb, Prkcz, Rac3a, Mapk10, Sos1, Fgf13b, Fgfr1b
	PDGF signaling pathway	Ehf, Mor, Arhgap1, Kras, Map2k2a, Shc2, Sos1, Rps6ka3b, Elf2b
Cerebellum specific proteins	Wnt signaling pathway	Csnk1g1, Ntla, Gnb3b, Ppardb, Sagb, Siah1, Wnt16, Wnt3, Wnt11, Ppp2ca, Prkchb, Wnt3a, Aes, Gng12a, Ppp2r5eb, Ppp3r1b, Prkcbb, Fzd6
	Gonadotropin releasing hormone receptor pathway	Nab1a, Gnb3b, Vcl, Cga, Jund, Bmpr1aa, Gnao1b, Gnb5b, Per1a, Prkcbb, Rela
	Inflammation mediated by chemokine and cytokine signaling pathways	Gnb3b, Jund, Arpc4, Arpc2, Gnao1b, Myh9a, Gng12a, Pdpk1b, Prkcbb, Rela, Arpc5la
Fin specific proteins	Angiogenesis	Mapk14a, Axin2, Pdgfaa, Hspb1
	Wnt signaling pathway	Mycl1b, Axin2, Wnt11r, Fzd10
	TGF-β signaling pathway	Mapk14a, Ndr1, Acvrl1
Retina specific proteins	Huntington disease pathway	Dync1li2, Dync1li1, Rhoq, Bdnf
	Alzheimer disease-presenilin pathway	Aph1b, Wnt4b, Furina
	Cytoskeletal regulation by Rho GTPase	Cfl1, Rock2a, Myo3a

The primary pathways for core proteins and organ-specific proteins were analyzed using PANTHER, whereby core proteins were identified by taking the intersection sets between each regenerative PPI network, while organ-specific proteins were identified through taking the relative complement set of each regenerative PPI network with respect to the other three PPI networks. The primary pathways for core proteins and organ-specific proteins were different from each other. The primary pathway for core proteins was TGF-β signaling pathway while the primary pathway was different for each organ; consisting of integrin signaling for the heart, Wnt signaling for the cerebellum, angiogenesis for the fin, and the Huntington disease pathway for the retina

In contrast, the primary pathways of the organ-specific proteins of the heart were the integrin signaling and the FGF signaling pathways. Integrins are trans-membrane receptors that act as bridges for cell-cell and cell-extracellular matrix (ECM) interactions that result in (transcriptional activation) responses such as regulation of the cell cycle, cell shape, and/or motility. FGF is a family of growth factors, with members involved in angiogenesis, wound healing, embryonic development, and various endocrine signaling pathways.

The primary pathway for cerebellum-specific proteins was identified as Wnt signaling. Wnt signaling has been recognized for its role in embryonic development control, including body axis patterning, cell fate specification, cell proliferation, and cell migration. It was reported that stimulation of Wnt signaling increases the number of neurogenic progenitors, which react to injury by proliferating and generating neuroblasts that migrate to the lesion site to repair damaged tissue in *zebrafish* cerebellum [26, 27].

The primary pathways of the organ-specific proteins for fin regeneration were angiogenesis and Wnt signaling. Angiogenesis is a normal and vital process in growth, development, wound healing, and in the formation of granulation tissue, whereas Wnt signaling has been reported to regulate the nerve reconstruction and blastema cell proliferation in fin regeneration experiments [28, 29].

The disease pathways for Huntington’s disease and Alzheimer’s disease were the primary pathways for the retina specific proteins. These pathways are related to neurons, indicating an influence on the regeneration of optic neurons. These pathways may play important roles during the regeneration processes, and further investigation of the *zebrafish* regeneration mechanism will be discussed in the Discussion section.

## Discussion

### The multi-step recalled-blastema-like formation model for investigating the role of core and organ-specific proteins

In this study, we propose a multi-step recalled-blastema-like formation model and attempt to classify the roles of the primary pathways both in core proteins and organ-specific proteins based on this model. Blastema is generally defined as a group of cells that gives rise to an organ or part in either normal development or regeneration. There is also existing research indicating brain also has blastema-like cells [30], which is the reason we named it “recalled-blastema-like formation”. The first step in regeneration is the injury response step. Generally, the injury response of cells in the wound environment includes cell surface changes that promote adhesion, migration, the formation of different cell/matrix interactions or endothelial shapes, and changes in permeability to enable leukocyte extravasation [31]. The second step is de-differentiation, where blastema can be derived from the de-differentiation of various functional cell types, such as skeletal muscle, dermis, and cartilage [32]. The next step is the recalled-blastema-like formation step. Broadly speaking, fibroblasts from the connective tissue migrate across the injured surface to meet at the center of the wound and then multiply to form a blastema. A blastema is a proliferative mass of morphologically similar cells that can develop into the structures lost after trauma. This characteristic is similar to embryonic development, and could be viewed as embryonic recall occurring in a regenerative process [33]; therefore we named this step the “recalled-blastema-like formation” step, from which the name of our multi-step model, the “recalled-blastema-like formation model”, is derived. The fourth step is the differentiation of recalled-blastema-like formation and pattern formation step. The model whereby blastema tissue differentiates into epithelial, chondrogenic, and osteogenic tissues is highly regarded in studies of wound repair [34]. Pattern formation is the reproducible generation of complex and self-regulating patterns, where Wnt signaling was proposed to play a dual role: as an activator during the process, and as an inhibitor after the process [35]. Although the datasets are limited to within five days after injury and while differentiation and pattern formation might not occur within these five days, we use this step to select candidate proteins for differentiation and pattern formation. The last step is the recovery step, which can be considered the termination step of regeneration.

### The TGF-β signaling pathway provides the vital signal needed to carry out a regenerative response

The TGF-β signaling pathway was identified as the primary pathway among the core proteins. Based on our analysis, we believe that the TGF-β signaling pathway provides one of vital signals needed to carry out the regenerative response, and it is involved in each step of our proposed recalled-blastema-like formation model for the regeneration of all four *zebrafish* organs. Members of the TGF-β family identified as core proteins include Smad7, Smurf2, Jun, Mapk1, Mapk3, Map3k7, Smad3a, Skib, Smad2, and Spaw.

**Fig. 2:**
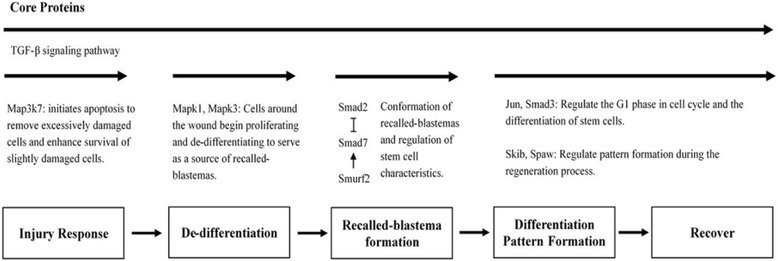
Recalled-blastema-like regeneration strategy in the TGF-β signaling pathway for core proteins. We proposed a multiple-step recalled-blastema-like formation model, including injury response, de-differentiation, recalled-blastema-like formation, differentiation, and pattern formation steps. The length of the arrows in the figure indicates duration of activities during the regeneration process. The TGF-β signaling pathway is the primary pathway identified for core proteins, including Smad7, Smurf2, Jun, Mapk1, Mapk3, Map3k7, Smad3a, Skib, Smad2, and Spaw. Map3k7 removes excessively damaged cells and also augments the survival of slightly damaged cells in the injury response step. In the second step, a source of recalled-blastema-like formation is produced by Mapk1 and Mapk3, which promote proliferation and de-differentiation in undamaged cells, which is induced by impaired tissue. In the third step, Smad2 and Smad7 act as antagonists while Smurf2 promotes the expression of Smad7. They coordinate the accumulation of stem cells and recalled-blastema-like formation to prepare for differentiation and pattern formation in the next step. Jun and Smad3 regulate the G1 phase of the cell cycle and mediate cell fate while Skib and Spaw regulate pattern formation. The last step can be viewed as termination of the regeneration process. TGF-β participates in each step and is predicted to serve as the vital signal needed to carry out the regeneration process

Map3k7, which controls cellular functions of transcriptional regulation and apoptosis, was identified as a core protein in all four regenerative core PPI networks. Map3k7 may be crucial to the regeneration of *zebrafish* organs due to its regulation of apoptosis and cell survival [25, 36]. Based on our result, Map3k7 might play a role in preventing wound deterioration through regulation of apoptosis and cell survival as part of the injury response during *zebrafish* organ regeneration (Fig. 2).

Amongst the regenerative core proteins identified through our regenerative PPI networks, we also observed two other mitogen-activated protein kinases: Mapk3 and Mapk1, also known as Erk1 and Erk2. As the downstream proteins of Map3k7, Mapk3 and Mapk1 participate in the regulation of a large variety of processes including cell adhesion, cell cycle progression, cell migration, cell survival, differentiation, metabolism, proliferation, and transcription [37]. This also indicates that Map3k7 may trigger the regeneration process through TGF-β signaling. The source of stem cells comes from the de-differentiation and proliferation of unimpaired cells, and Mapk1 and Mapk3 have been reported to support the regulation of cell proliferation during liver and nerve regeneration [38, 39]. They have also been reported to mediate de-differentiation of hepatocytes through the epithelial-mesenchymal transition [40]. Both Mapk1 and Mapk3 were observed as core proteins, indicating that, in *zebrafish* organ regeneration, Mapk1 and Mapk3 may play a role in blastema de-differentiation to develop into the structures lost after trauma (Fig. 2).

In the TGF-β signaling pathway, Smad2 and Smad7 are antagonists, and Smurf2 can enhance the expression of Smad7. Smad2 expression within the blastema was increased during tail regeneration in the leopard gecko, while Smad7 regulates blastema formation at the early stage of *zebrafish* fin regeneration, indicating indispensable roles for Smad2 and Smad7 in the *zebrafish* blastema [41, 42]. It was also reported that mice lacking exon 1 of the Smad7 gene exhibited reduced neural stem and progenitor cell quiescence and proliferation in the lateral ventricles, indicating that Smad7 regulates stem cell activity [43]. Thus, Smad7 may act as a regulator for *zebrafish* recalled-blastema-like formation alongside the antagonist Smad2 and enhancer Smurf2; and we speculate that Smad2, Smad7, and Smurf2 play a role in coordinating the formation of the recalled-blastema-like during *zebrafish* organ regeneration.

Smad3 and Jun have been reported to participate in the regulation of G1 to S phase cell cycle transitions by maintaining sufficient cyclin D1 kinase activity [44, 45]. Additionally, Smad3 has been reported to co-mediate and control the differentiation of stem cells into T-cells, myofibroblasts, oligodendrocyte progenitors, and others [46]. It was proposed that the regulation of the G1 phase of the cell cycle might affect cell type during the differentiation of human embryonic stem cells [47]. Similarly, the regulation of the G1 phase by Jun indicated that Jun might regulate differentiation ability in *zebrafish.* Consequently, we anticipate that Smad3 and Jun may participate in mediating the differentiation of the recalled-blastema-like into the proper cell types during *zebrafish* regeneration (Fig. 2).

Skib and Spaw were also identified as core proteins based on our comparisons. We speculate that the biological function of these two proteins might be involved in pattern formation as part of the underlying mechanism for *zebrafish* organ regeneration processes. In previous experiments, overexpression of Skib resulted in a dorsalized phenotype while inhibition of Skib led to the loss of head structures during the development of *zebrafish* embryos, demonstrating that Skib can regulate pattern formation [48, 49]. Furthermore, an experiment on the asymmetric development of cardiac morphogenesis in *zebrafish* showed that Spaw is required for a correct left-and-right asymmetry pattern for the migration of cardiac progenitor cells [50]. Increased expression of Spaw also results in looping defects in the *zebrafish* heart [51]. Despite the limitation of the time course microarray data to within three days, these studies provide evidence of the potential roles played by Spaw and Skip in pattern formation by progenitor cells (Fig. 2).

**Fig. 3:**
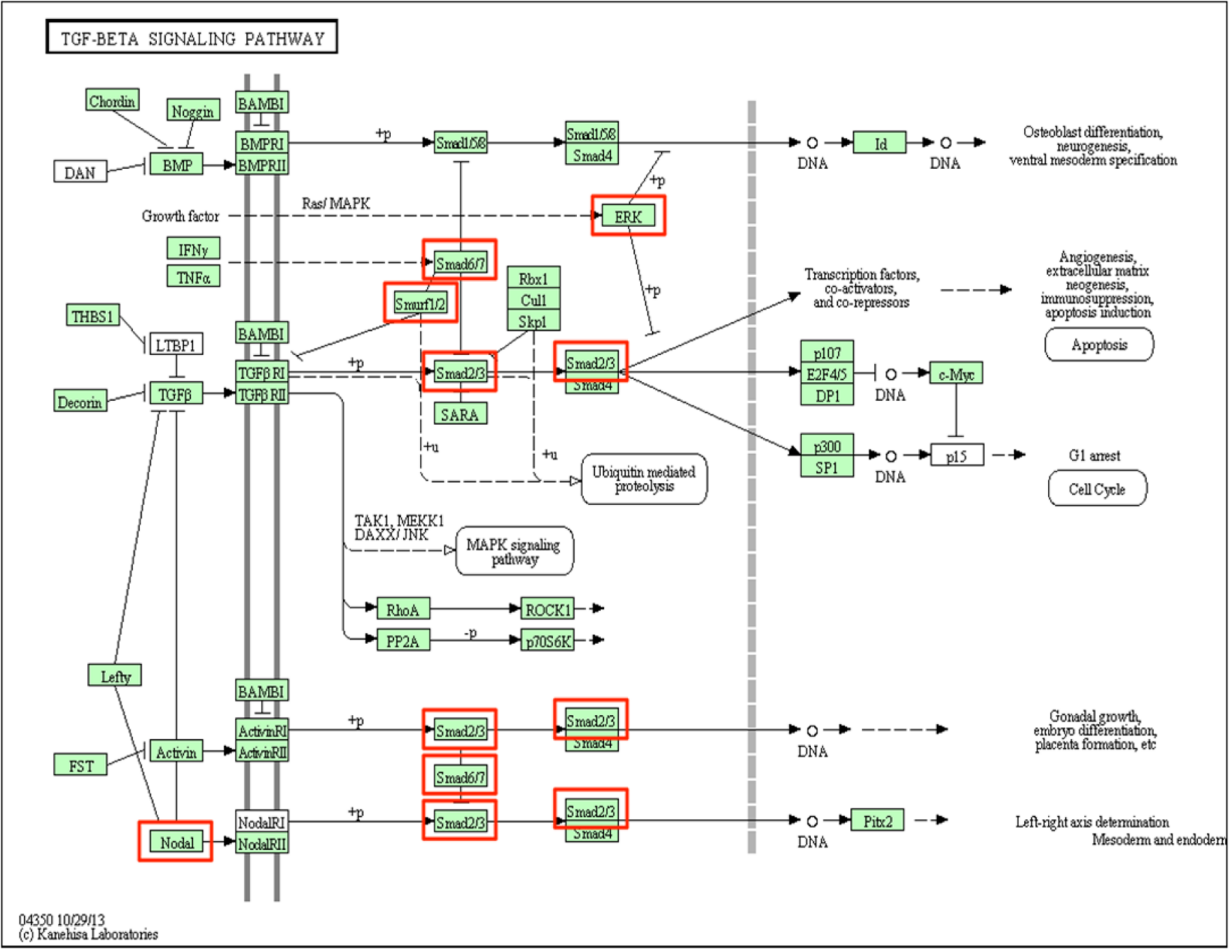
KEGG protein distribution of TGF-β pathway proteins observed among the core proteins. The identified TGF-β signaling proteins were mapped to KEGG. These proteins act as the bridge in the TGF-β signaling pathway, and show significant association with multiple functions in the TGF-β pathway, including osteoblast differentiation, neurogenesis, ventral mesoderm specification, induction of apoptosis, regulation of G1 arrest, and left-right axis determination

The regenerative functions for these ten proteins indicate the role of TGF-β in the recalled-blastema-like formation during the regeneration process. The distribution of the ten proteins in the TGF-β signaling pathway of the Kyoto Encyclopedia of Genes and Genomes (KEGG) [52] depicted in Fig. 3 shows a significant association with the biological functions for TGF-β, including osteoblast differentiation, neurogenesis, ventral mesoderm specification, induction of apoptosis, regulation of G1 arrest, and left-right axis determination. Based on the analysis above, we believe that the TGF-β signaling pathway plays a crucial role in the regeneration of all four *zebrafish* organs and may provide the common, vital signal needed to carry out a regeneration response drive for *zebrafish* organs. However, we also believe that other important mechanisms exist, which are specific to particular organs during the *zebrafish* regeneration process. This topic will be discussed in the following sections.

### Integrins, PDGF, and FGF regulate binding affinity of fibrin and fibrinogen for rapid hemostasis during zebrafish heart regeneration

**Fig. 4:**
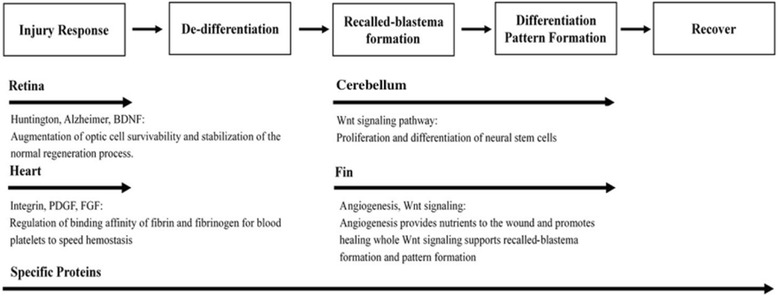
Regeneration strategy of proteins specific to the heart, cerebellum, fin, and retina in the recalled-blastema-like regenerative model. In heart-specific proteins, Integrin, PDGF, and FGF co-regulate binding affinity of fibrin and fibrinogen for platelets to speed hemostasis in the injury response step. In cerebellum-specific pathways, Wnt signaling promotes formation of the recalled-blastema-like formation via Wnt3 and participates in the regulation of neuronal differentiation and cerebellum structure via Wnt3a and Ppp2ca. For fin-specific proteins, it was observed that angiogenesis provides nutrition to promote the recalled-blastema-like formation and pattern formation through Wnt signaling. In retina-specific proteins, Bdnf enhances optic cell survivability and stabilizes the regeneration process. These proteins may trigger differentiation and de-differentiation processes for the zebrafish regeneration process

The primary pathways of the organ-specific proteins, which were identified in our constructed heart regenerative PPI network and analyzed by PANTHER, are listed in Table 2. Since there is very little information regarding the gonadotropin releasing hormone receptor pathway (which was one of the primary pathway) in heart regeneration studies, we will focus our discussion on the second primary pathway. Another primary pathway enriched in the heart specific proteins was an integrin signaling pathway with 13 proteins: Parvaa, Rras, Map2k2a, Kras, Lamb1a, Rac3a, Tln1, Arl1, Mapk10, Vasp, Lama5, Sos1, and Bcar1. One of prominent functions of integrins is to regulate the binding affinity of fibrin and fibrinogen for blood platelets. At the same time, the fibroblast growth factor (FGF) signaling pathway and platelet-derived growth factor (PDGF) signaling pathway were also observed among the organ-specific proteins for the heart regeneration process in our PPI network. FGF and its receptors participate in the regulation of cell differentiation, proliferation, angiogenesis, and survival [53], while the PDGF signaling pathway controls the binding of platelets to fibrin to form clots and stop bleeding [54]. It has been proposed that FGF signaling interacts directly with integrin signaling [55], such that PDGF and FGF may co-regulate binding affinity for fibrin and fibrinogen to allow for rapid hemostasis through the mediation of integrins during heart injury. This might serve as the primary regenerative strategy for heart regeneration during the injury response step (Fig. 4).

### The Wnt signaling pathway improves proliferation and differentiation of neural stem cells in cerebellum

The Wnt signaling pathway was identified to be the main pathway for cerebellum-specific proteins including Wnt3, Wnt3a, Wnt11, Wnt16, Siah1, Ppp2ca, Gng12a, and Fzd6 (Table 2). Wnt3 expression is positively correlated with the proliferation of neural stem cells, promoting neuron proliferation [56]. The promotion of neural stem cell proliferation by Wnt3 indicates the formation of the recalled-blastema-like, and can be classified in the recalled-blastema-like formation step of the regenerative model, as shown in Fig. 4. Siah1 has been reported to be involved in the CNS injury response [57]. Wnt3a is required for the formation of cerebellum structures and participates in the regulation of cerebellum structure, indicating that it has a role in pattern formation in the proliferating blastema, while Ppp2ca participates in the differentiation of neural stem cells [56, 58]. These cerebellum-specific proteins can be classified into the differentiation and pattern formation steps of the recalled-blastema-like regenerative model (Fig. 4), and provide potential targets for the investigation into cerebellum regeneration. These neuron-related studies support the possibility that the Wnt signaling pathway plays an essential role in cerebellum regeneration. Overall, the focus of the regeneration strategy of the cerebellum may be on the proliferation of neural stem cells and the following differentiation and pattern formation of injured tissue through Wnt signaling.

### Angiogenesis provides nutrients and promotes the healing of wounds while Wnt signaling supports the recalled-blastema-like formation and pattern formation during zebrafish fin regeneration

In the case of *zebrafish* fin regeneration, the primary pathway enriched in organ-specific proteins was angiogenesis, and this included Mapk14a, Axin2, Pdgfaa, and Hspb1 (See Table 2). One of the angiogenesis proteins, Mapk14a, known as p38a, has been reported to regulate the differentiation of myoblasts, prevent fibrosis, and to improve and repair muscles [59]. Skeletal muscle differentiation was shown to be mediated by both the muscle-specific transcription factor myogenin and Mapk14a [60]. Without Mapk14a signaling, myogenin may lead to the down-regulation of genes involved in cell cycle progression [60]. Another identified organ-specific protein, Axin2, is involved in cell differentiation and the regulation of osteoblast differentiation. Additionally, Pdgfaa was shown to participate in the positive regulation of cell proliferation and migration [61]. It has been reported that the release of Pdgfaa greatly promotes the effective recruitment of human mesenchymal stem cells [62]. Overall, Mapk14a and Axin2 co-regulate the regeneration of bone and muscle, while Pdgfaa accelerates these processes through the recruitment of stem cells. These proteins can be classified in the recalled-blastema-like formation and pattern formation steps of our recalled-blastema-like regeneration model (Fig. 4).

### Bdnf augments the survivability of optic cells and stabilizes the retina regeneration process

The primary pathway of organ-specific proteins in the retina PPI network is the Huntington’s disease pathway. Bdnf, which is a member of neurotrophin growth factor family and part of the Huntington’s disease pathway, helps support the survival of existing neurons as well as encouraging growth and differentiation in neurons and synapses, both in the CNS and PNS [63, 64]. An experiment into the axonal regeneration of retinal ganglion cells indicated that Bdnf promotes short-term cell survival after optic nerve injury [65], indicating the essential role played by Bdnf in retinal regeneration. Another pathway enriched in the retina-specific proteins was the Alzheimer’s disease-presenilin pathway. Both Alzheimer’s disease and Huntington’s disease are neuron-related diseases and the expression of neurodegenerative disease-related proteins may represent a progressive loss of structure or function in neurons, such as neuron death [8]. Given that the upregulation of these neurodegenerative pathways might have a negative effect on the neuron regeneration process and cause secondary damage to neural systems, the activation of Bdnf in retina-specific proteins may indicate the importance of preventing such secondary damage in neurons during *zebrafish* retina regeneration. It has been reported that Bdnf promotes and stabilizes the morphological maturation of retinal axonal arbors by influencing both the synapses and axon branches, indicating that Bdnf also helps stabilize the retina regeneration process [66]. Consequently, the primary regenerative strategy of retina-specific proteins may be to prevent secondary damage to the retinal neurons and to augment the survival of optic cells during the injury response step (Fig. 4).

### Bioinformatics insights and a regenerative strategy inferred from core and organ-specific proteins of the regenerative processes of the organs and appendages of zebrafish

In summary, TGF-β signaling, which is the primary pathway observed in the core proteins, provides the vital signal for the regeneration of all four organs. TGF-β signaling participates in each stage of the recalled-blastema-like formation model and plays various roles during the regeneration process. On the other hand, Mapk3k7 is the upstream of Mapk1 and Mapk3 in the injury response step, and it may serve as the primary trigger for TGF-β signaling in the regeneration process. For organ-specific proteins, we also observed rapid hemostasis through the co-regulation of integrins, PDGF, and FGF in heart-specific proteins during the injury response step. For cerebellum-specific proteins, Wnt signaling participates in neural stem cell proliferation through mediation by Wnt3 and differentiation by Ppp2ca. For fin-specific proteins, both Mapk14a and Axin2 regulate the differentiation of myoblasts and osteoblasts. By conducting the analysis using our recalled-blastema-like formation model, we provided a model to explain the proteins, interaction and their roles in regeneration process of *zebrafish*. Moreover, these proteins could be used as targets for further study into the underlying mechanism of *zebrafish* organ regeneration.

## Methods

### Zebrafish husbandry & ethics statement

*Zebrafish* were maintained based on the guidelines described in the Zebrafish Book. *Zebrafish* were reared at a density of about 50 fish per 10 l of water in individual tanks connected to a circulating water system (AZOO, Taiwan); the water temperature was maintained at 28.5 °C.

Experimental procedures were performed in accordance with Institutional Animal Care and Use Committee (IACUC) number 09808 and approved by the Committee for the Use of Laboratory Animals at National Tsing-Hua University.

### Ventricular resection

**Fig. 5:**
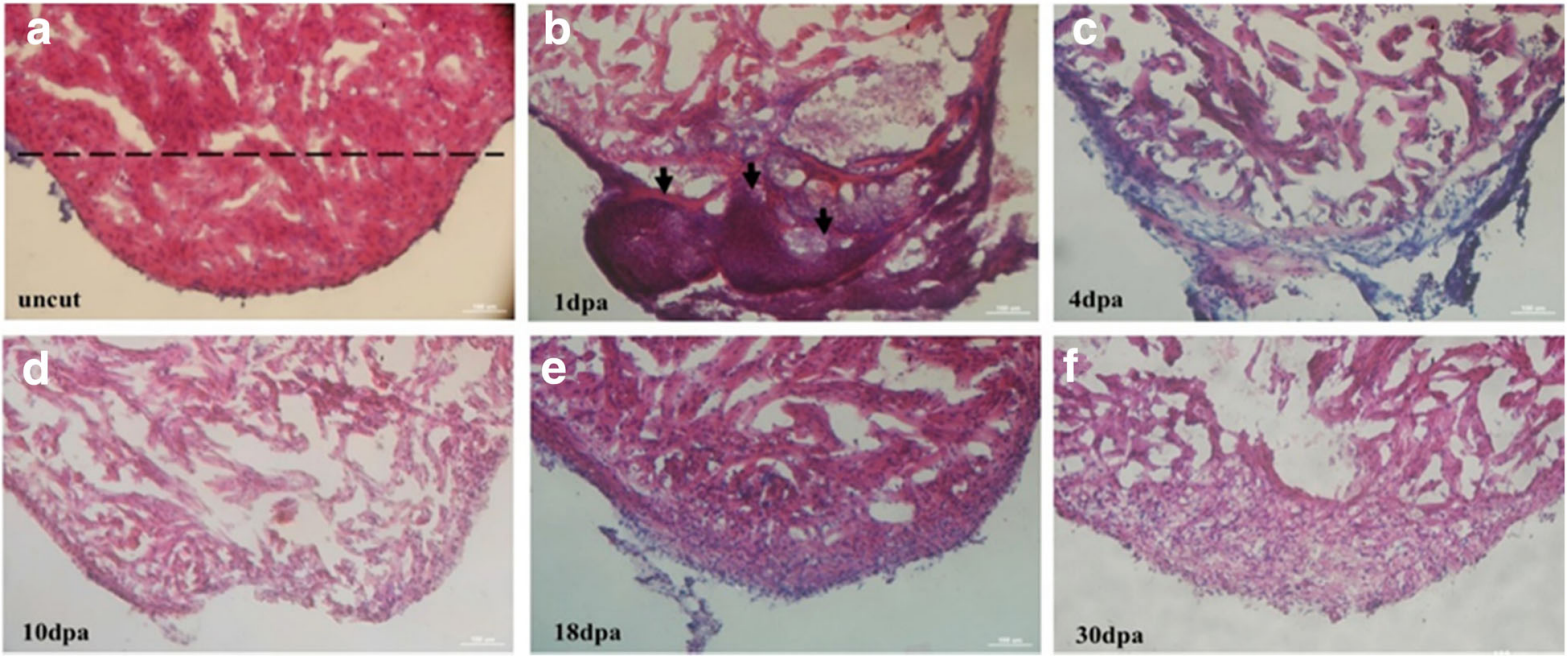
Different phenotypes of injured zebrafish heart. The stages of heart regeneration after amputation arranged by day, including (**a**) uncut, (**b**) 1 dpa, (**c**) 4 dpa, (**d**) 10 dpa, (**e**) 18 dpa, and (**f**) 30 dpa. The dashed line represents the line of amputation during surgery and is about 10–20% of the ventricular apex. Scar bar at the lower right corner indicates 100 uM in length

Test subjects were first anesthetized using a mixture of MS-222 (Sigma-Aldrich) and isoflurane (Baxter), to allow for faster recovery and achieve a higher success rate after surgery. Micro-scissors were used to create a small incision and then quickly push out the beating heart; about 10%~ 20% of the ventricular apex was amputated before the fish were returned to water for recovery (Fig. 5).

### Heart collections

For the heart collections, total RNA were extracted from the ventricles using Trizol reagent and dissolved in reagent-grade water (Sigma-Aldrich). The mRNA samples were then sent to a specialized commercial microarray service company (Welgene Biotech, Taiwan) for subsequent sample processing and data collection. Co-respondent time point contains 10 hearts from the test subjects to provide an averaged gene expression profile of the group. All time points were analyzed systematically as described in the text.

### Microarray and data analysis

Three sets of 10 fish were used. Each fish in each group was injured by ventricular resection from day 0 to 28, respectively. These injured fish were collected at 0, 0.25, 1, 3, 6, 10, 15, 21, 28 dpi (day post injury). 1.65 μg of Cy3 cRNA for *zebrafish* array was fragmented to an average size of about 50–100 nucleotides by incubation with fragmentation buffer at 60 °C for 30 min. Each time points contain two biological repeats.

## Conclusions

Regenerative medicine may one day allow us to replace, engineer or regenerate human cells, tissues or organs to restore or establish normal function. With the help of high-throughput data and systems biology methodology, we hope to unravel the fundamental mechanism of organ regeneration for zebrafish, which may lead to further breakthroughs in regenerative medicine. In this study, we use regression dynamic model to identify interaction ability of PPIs to construct four regenerative PPI networks. Dynamic regenerative PPI network construction captures activated pathways based on experiment data through applying AIC to select significant PPIs. We use the intersection set between four zebrafish regenerative PPI networks to identify core PPI proteins and the corresponding core PPI networks for each organ. Furthermore, we use the difference set to identify crucial proteins that are specific to a particular organ regeneration. After applying pathway analysis on core and specific proteins, we identified TGF-β signaling for core proteins, integrins for heart specific proteins, Wnt signaling for brain specific proteins, angiogenesis for fin specific proteins, and Bdnf for retina specific proteins. Furthermore, we proposed a multi-step recalled-blastema-like formation model to classify these pathways to uncover the underlying mechanism for zebrafish organ regeneration.

In this study, microarray data for all four zebrafish organ regeneration were used to constructed regenerative PPI networks and to extract core and specific proteins. The accuracy of our constructed regenerative PPI networks can be further improved if more data are available. For instance, protein expressions used here are overlaid by mRNA expression. If high-throughput proteomic data on zebrafish organ regeneration were made available in the future, we will be able to construct regenerative PPI networks with improved accuracy. Furthermore, PPI information for zebrafish is derived from ortholog to human PPI. The integrated zebrafish PPI information based on real experiments can also be a crucial improvement on the construction of regenerative PPI networks. In addition, next generation sequence (NGS) would provide more comprehensive information such as description of the locations of histone post-translational modifications and DNA methylation genome-wide. It also has higher resolution for visualizing in the genome epigenetic marks. An integrated cellular network of transcription regulations and PPI networks based on NGS and high-throughput proteomics can better equip us for further investigation on zebrafish organ regeneration.
